# Stakeholder perspectives on surveillance of physical activity and monitoring and evaluation of interventions in Saudi Arabia

**DOI:** 10.1186/s12889-025-22631-5

**Published:** 2025-04-12

**Authors:** Hazzaa M. Al-Hazzaa, Zara Shubber, Mezna AlMarzooqi, Abdullah F. Alghannam, Shaima A. Alothman, Yousef AlQurashi, Reem AlAhmed, Ahmad Hegazi, Sameh El-Saharty, Reem F. Alsukait, Severin Rakic, Volkan Cetinkaya, Saleh A. Alqahtani

**Affiliations:** 1 Lifestyle and Health Research Center, Health Sciences Research Center, Princess Nourah Bint Abdulrahman University, Riyadh, Saudi Arabia; 2 School of Sport Sciences, University of Jordan, Amman, Jordan; 3 World Bank Group, Washington, DC USA; 4 Leaders Development Institute, Ministry of Sport, Riyadh, Saudi Arabia; 5 Respiratory Care Department, College of Applied Medical Sciences, Imam Abdulrahman Bin Faisal University, Dammam, Saudi Arabia; 6 Liver, Digestive, and Lifestyle Health Research Section, and Biostatistics, Epidemiology and Scientific Computing Department (BESC), King Faisal Specialist Hospital & Research Center, Riyadh, Saudi Arabia; 7 Liver, Digestive, and Lifestyle Health Research Section, and Organ Transplant Center of Excellence, King Faisal Specialist Hospital and Research Center, Riyadh, Saudi Arabia; 8 Division of Gastroenterology and Hepatology, Weill Cornell Medicine, New York, NY USA

**Keywords:** Physical activity, Monitoring, Evaluation, Surveillance, Saudi Arabia, Vision 2030

## Abstract

**Background:**

Promoting physical activity (PA) has been one of the key strategic priorities in Saudi Arabia. At the population level, policies targeting PA can potentially enhance health and well-being. One of the objectives of the Quality-of-Life Program within the Saudi Vision 2030 is to increase public participation in sports and PA. The present paper describes the outcomes of a participatory workshop focusing on stakeholder perceptions of the current state of surveillance of PA and monitoring and evaluation (M&E) of PA programs and initiatives.

**Methods:**

A workshop was organized fostering a discussion between 29 key attendees from 8 different government entities and other organizations representing the policymakers, researchers, planners, and implementers of programs that focus on promoting or evaluating PA. Stakeholder perceptions on the current state of M&E and surveillance of PA were gathered. A list of actions generated from the identified gaps in the established programs was created, and areas of opportunities for collaboration were identified.

**Results:**

The stakeholder discussion highlighted key themes related to PA surveillance, M&E, including the perceived strengths and challenges and the key priority actions to address the gaps. Additional important steps include creating a national strategy and action plan for PA, with M&E framework that comprises key goals, targets, indicators, unified data collection methods, and guidance on ensuring coordinated and wide dissemination.

**Conclusion:**

M&E of PA in Saudi Arabia requires a multi-stakeholder, multi-sectoral approach, facilitated by creating a specialized governance and leadership committee. Some recommendations include establishing a multi-sectoral structure to lead PA in Saudi Arabia and developing mechanisms for sharing experiences and knowledge. Policymakers need to embrace such an agenda and advance discussions on the scope and structure of M&E and surveillance systems for PA.

**Supplementary Information:**

The online version contains supplementary material available at 10.1186/s12889-025-22631-5.

## Background

Noncommunicable diseases (NCDs), including cardiovascular diseases, cancers, diabetes, and chronic respiratory diseases, are the leading causes of mortality both globally and locally [1, 2]. The World Health Organization (WHO) defines PA as any bodily movement produced by large (skeletal) muscles that expends energy above the resting value and recommends at least 150 min of moderate intensity activity or 75 min of vigorous intensity activity per week for adults [3]. Inadequate PA is regarded as a modifiable risk factor for NCDs and a major public health concern. Engaging in regular PA is an effective strategy to improve individual health and the overall well-being of a population [1]. It has been shown to help protect and manage several NCDs, including heart disease, stroke, diabetes, hypertension, and some cancers [3]. It also helps maintain a healthy body weight and improve mental health and overall well-being [3]. PA can take many forms, including walking, running, cycling, and any form of sport, play, or household activity, and can be done at any intensity, which is versatile enough to include some form of activity suitable for everyone.

The need for increasing PA and reducing sedentary behaviors is high. The level of PA among Saudi adults ranges from 50 to 90% [4–6]. A recent survey by the General Authority for Statistics (GASTAT) revealed that about 30% of the resident population engaged in moderate PA for at least 150 min per week [7]. As part of the Quality-of-Life Program of the Saudi Vision 2030 [8], the country aims to achieve an increase in the number of individuals who weekly engage in sports and PA and provide more good quality urban public open spaces suitable for PA. The need for policies to increase population levels of PA appears most important [9]. A report from the WHO Regional Office for the Eastern Mediterranean on PA promotion included a policy mapping assignment on national policy and action on PA in all regional member states [10]. The report stressed the importance of partnerships between various ministries in the Gulf states to promote PA [10].

Saudi Arabia has recently developed initiatives to promote health-enhancing PA [11]. Policies targeting PA can potentially enhance health and well-being at the population level [12]. Programs that promote PA behavioral change are comprehensive, involve multiple stakeholders, and target multiple population groups. Additionally, they are often costly and require time to implement and achieve results. Therefore, these programs must be closely monitored and evaluated to generate evidence that explains what works and in which settings. Careful planning, M&E of PA programs provide higher quality, practice-based evidence that can serve as a guide for designing and implementing additional programs [13, 14]. This helps promote national- and sub-national-level efforts by sharing knowledge and disseminating experience.

A recent review of national health-enhancing PA policies in Saudi Arabia identified 44 policies across five sectors (health, sports, education, tourism, and urban environment) between 2016 and 2022 [11]. While 30 policies were reported to have an evaluation component, the definition and approach to evaluations varied. Many sources were also found to be used to report on PA trends, including the 2019 World Health Survey-Saudi Arabia (WHS-SA) implemented by the Ministry of Health (MoH) [15], national-level surveys, including the GASTAT’s Family Health Survey and Sports Survey [16], and the Nielsen Global Health and Wellness Survey Annually used by the Saudi Sports for All Federation [11]. The WHS-SA, for example, intended to provide the status of relevant health-related indicators and WHO metrics, including behavioral indicators such as PA levels and dietary habits. It is a monitoring system expected to assist policymakers and program managers in designing, evaluating and monitoring programs and strategies [15]. It is well-recognized that policies can impact population levels of PA; however, there is a further need for research related to PA policies.

To determine the perspectives of key stakeholders, we designed and executed a workshop on surveillance and M&E of PA in Saudi Arabia. The aim of the workshop was to gather perspectives and encourage active dialogue between key stakeholders, such as policymakers, researchers, planners, and implementers of PA programs in Saudi Arabia. This paper reports on the outcomes of the workshop and highlights the key actions that need to be implemented to achieve the goals set out by the Saudi Vision 2030.

## Methods

On May 4, 2023, a full-day workshop was organized in Riyadh as a collaboration between King Faisal Specialist Hospital and Research Center (KFSHRC) and the World Bank to understand how Saudi Arabia can strengthen its systems and capacities in the surveillance of PA and M&E of PA interventions. We aimed to learn from the attendees’ experiences and knowledge, while balancing plenary and small-group discussions. A detailed workshop program is provided in Additional File 1.

### Attendees

The workshop was structured to encourage discussion among attendees with diverse backgrounds. The selection of attendees was twofold; some were invited after their prior participation in the “Comprehensive Review of PA Policies and Initiatives in Saudi Arabia 2016–2022” [11], while others were selected based on the relevance of their work/organizations’ mandates. Given the number of confirmed attendees, they were pre-assigned to three breakout groups, based on their organizational affiliation, with the aim of achieving diversity among the groups in terms of sector, gender, and seniority. Each small group contained representatives from the organizations that could ensure a diverse discussion and increase the visibility of projects that might have been siloed from each other. A facilitator was assigned for each breakout group to guide the discussion and prepare a summary of the main discussion points and views of the attendees. Three facilitators were selected on the basis of their experience in group facilitation, previous experience with qualitative research, and familiarity with PA promotion activities in Saudi Arabia.

### Program

Preparation for the workshop included developing facilitator guides and reporting templates to facilitate the reporting back to the group. Plenary sessions were held in the larger hall, while breakouts were split between several spaces. The structure of the workshop enabled the maximum time to be spent in discussion with the attendees. However, some pre-planned content was included at beginning of the program in order to set the stage for the discussion and explain to the attendees how the workshop would flow (Table 1). Two key questions were discussed during the breakout sessions: (i) what is the current status of PA surveillance and M&E of PA programs - what are strengths and challenges? and (ii) what priority actions were needed? How can stakeholders collaborate to strengthen PA surveillance and M&E?

**Table 1:** Summary of workshop program

Session	Content
Opening remarks	The opening remarks included a self-introduction of the attendees as well as introducing the agenda for the day and the goals of the workshop.
Setting the stage	This session included a presentation on the findings from the study “Comprehensive Review of PA Policies and Initiatives in Saudi Arabia 2016–2022” that identified how evaluations were defined and approached across five sectors (health, sports, education, tourism, and urban environment) [11]. All participants attend the session.
Breakout sessions and plenaries	*Breakout session 1: What is the current status of PA surveillance and M&E of PA programs?*: The session started with a round of organizational introduction, which included recent projects relevant to PA promotion that attendees were working on. The strengths, weaknesses and challenges are then discussed. *Plenary 1*: Each group presented the key takeaways from their breakout session to the plenary. Data were cross-checked, points of agreement were identified, unique perspectives of the groups were discussed, and consensus was reached among the groups. *Breakout session 2: Priority actions and stakeholders to strengthen PA surveillance and M&E in Saudi Arabia*: The session built on the previously discussed strengths and challenges, focusing on future actions and improvements, including strategies to support collaboration. *Plenary 2*: Each group presented the key takeaways from their breakout session to the plenary. Data were cross-checked, points of agreement were identified, unique perspectives of the groups were discussed, and consensus was reached among the groups.
Closing remarks	Reviewing what was achieved during the workshop, confirming consensus among the groups, and discussing potential next steps.

### Data collection and analysis

The qualitative data used to develop the paper were generated through the two breakout group discussions. The data were collected through notetaking by the facilitators and validated by attendees at the end of each breakout session. Data was collected on Microsoft PowerPoint and paper flip boards. The discussions were not recorded, and no direct quotes were captured from the attendees. Consensus on the captured points was reached by jointly validating the summary data and allowing for time and input from all attendees in the breakout groups. Data were cross-checked between the breakout groups during plenary sessions and consensuses were reached among the groups. Notes from each group were summarized and first analyzed separately by the facilitators in order to identify the themes covered in each breakout group (Additional File 2). In the next step, themes emerging from the three groups were cross-checked for recurring themes and points of agreement, combined, and jointly analyzed by the authors before an exhaustive list of takeaways from the workshop was compiled.

## Results

The workshop was attended by 29 participants who represented eight organizations, including line ministries, national health institutions, and key regional, national, and local organizations implementing PA promotion programs. Each group had a separate breakout discussion. The benefit of breakout groups’ diversity was evident from the beginning of the sessions; after a round of introduction, the discussion quickly gravitated towards natural connections between projects the attendees were working on or knew of. The facilitators guided the discussion, ensured that each attendee had an opportunity to contribute, and assured that the perspective of each organization was well represented in the breakout groups.

### What works in physical activity surveillance, monitoring and evaluation?

There are multiple examples of programs that collected good data on PA and targeted different populations, either with a specific focus on PA or with PA as a component of a larger program. The actors implementing these programs include health authorities, academic institutions like Princess Nourah bint Abdulrahman University, Riyadh, research centers like KFSHRC, and organizations like Saudi Sports for All Federation (SFA) [17]. In addition, there is an existing collaboration between national and international organizations, like the SFA and WHO, the KFSHRC, and the World Bank, all guided by the leadership’s goal in Saudi Vision 2030 [10]. All these initiatives collect varied and precise data, including disaggregated information by geography and age, and utilize new innovative approaches like creating experience maps or wearable devices and apps for data collection.

Nourah Health includes a health-promoting component that takes place throughout the academic year, in addition to data collection for a cohort study that includes staff and students annually. The data collected is an annual assessment of the health promotion activities conducted over the year [11]. Other examples include the MoH implementing a community-based program that assesses the determinants of health within cities, including the indirect assessment of PA. The KFSHRC is working with Johnson & Johnson on an employee wellness program that covers 16,000 employees and their families and provides incentives for healthy behavior in several domains, including PA.

Another example of a successful PA promotional program introduced in Saudi Arabia was the SFA’s after-school Neighborhood Clubs (NC) program launched in 2022 in collaboration with the Ministry of Education [17]. The program began at 13 locations and was expanded to 32 locations in 22 cities in 2023. It includes activities such as football, volleyball, basketball, gymnastics, yoga, dance, and others. It is operated at schools and is open to the public, although the majority of participants are school-age youth.

### Challenges with monitoring and evaluation of physical activity programs

The discussion highlighted the need for further collaboration between stakeholders and the alignment efforts in evaluating PA. This was accompanied by the utilization of different methodologies and approaches, which challenged the comparability of the programs and their results. Furthermore, different monitoring approaches lead to the collection of different data variables, resulting in independent data sets often siloed from other stakeholders. Some of the monitoring results remain unpublished, which hinders visibility of what has been done and creates a challenge to program’s sustainability. Another point raised was the need for a centralized knowledge hub that would compile the lessons learned from using different methodologies and insights that could guide future projects and the general need for deliberate dissemination of knowledge. Furthermore, there is a need for coordination in the impact evaluation tools and definitions of PA used by different stakeholders, in addition to the need for guidelines that support the consolidation of efforts for evaluating the PA programs. The unaligned efforts result in multiple small programs targeting small cohorts instead of more powerful long-term and large-scale assessments that can be potentially repeated at regular intervals to assess progress toward clear and long-term goals.

Second point raised during this part of discussion is the need for a clear governance for PA-related programs, including M&E. The attendees highlighted the importance of a centralized governing body, that could engage and collaborate with implementers nationwide and act as a clear leader in PA strategy development. The governing body could guide generalized and population-specific efforts while tracking goals and helping to set national and regional policies specific to PA. This would strengthen the engagement of stakeholders from different sectors, leading to a stronger involvement from the private sector and nongovernmental organizations, both valuable allies and relevant stakeholders to PA.

### Priority actions

The importance of addressing the need for high-level resources that govern PA strategy and M&E was identified as one of the main priorities. This was expressed in two aspects: (1) establishing a specialized governing body and (2) creating a national multi-sectoral framework for M&E.

The specialized governing body would nationally represent different sectors and organizations involved in PA, thus including all stakeholder perspectives. Its function would be to oversee goal setting and be responsible for creating and reviewing high-level policies that aim to govern PA in Saudi Arabia. Furthermore, the participants recommended the creation of a framework to guide M&E efforts, including stricter requirements for M&E frameworks for any new PA programs, along with clear evaluation mechanisms from the inception stage. A good example was drawn from the National Committee for Tobacco Control, which creates and reviews guidelines for improving tobacco cessation services that align with global frameworks, like the WHO Framework Convention for Tobacco Control [18]. Another function suggested by the groups for the governing body was establishing a national surveillance system to collect surveillance data on wider determinants of PA, such as weather, terrain, and urban planning. This data could be segmented by age groups, urban/rural settings, and different population groups in Saudi Arabia. Qualitative research projects would then be guided by these segmentations to help with understanding the barriers that specific groups face.

Another priority noted by all groups was addressing the need to unify data collection. This would involve identifying and setting specific indicators for PA, which can then be monitored either on their own through PA-specific programs and tools or as part of a wider survey on risk factors for NCDs.

Furthermore, communication of data and knowledge was prioritized by several groups as well. This was expressed in response to a need for improved data accessibility in order to display and share progress on national PA goals. A suggestion was to create a public online dashboard showcasing the collected data and the trends resulting from its analysis. Organizing an annual Saudi conference on PA is another method of facilitating communication between stakeholders and sharing knowledge and experiences. This discussion also resulted in recommendations for establishing sustainable funding streams for PA-related projects and exploring broader and more in-depth use of innovative methods of monitoring and promoting PA.

### Key stakeholders

The workshop uncovered the desire to build stronger connections between the stakeholders who attended it and highlighted the importance of communication between sectors. The attendees represented key stakeholders and collectively represented some sectors of interest in Saudi Arabia. Throughout the workshop, the attendees exchanged information about current projects, potential collaborations, and contact information. Attendees also discussed additional institutions and sectors that they would like to join the conversation about PA (Additional File 3). These additional stakeholders are aligned with the WHO Global Action Plan on PA 2018–2030 [13].

One function of the governing body would be to address the need for guidance on approaching and engaging with additional stakeholders, including nongovernmental organizations or the private sector.

## Discussion

Stakeholder engagement is increasingly gaining a grip in many fields, including healthcare policy [19, 20]. In a recent study reporting on a workshop intended to develop a PA agenda based on community-identified priorities and understand factors impacting PA participation in communities across northern British Columbia, the two-day event was shown to be successful at achieving its objectives and engaged a diverse group of multi-sectorial stakeholders from a broad geographic region [21]. In addition, according to a report published by WHO on the global action plan to promote PA, stakeholders should develop and support national and or subnational policy implementation by multi-sectoral partnership, including monitoring of such national policy as well as strengthening governance systems and accountability [13].

The collective knowledge of our workshop attendees resulted in a rich discussion, with multiple points of alignment in strengths, perceived gaps, and priorities for the way forward. The overall outcomes of the discussion showed multiple PA M&E priorities from the attendees’ points of view.

### Prominent emerging themes

#### Thoughtful, successful, with a need for further alignment

Saudi Arabia already has multiple examples of thoughtful and successful programs and initiatives that target promoting and examining PA in certain populations [11]. However, multi-sectoral engagement and governance structures for PA promotion are needed to help aid in the overlap between initiatives by different stakeholders and reduce duplication of effort. These efforts, although good on their own, offer a patchwork of pieces with the same intention but result in non-standardized data on different indicators and target different communities. Thus, the resulting data cannot be compared. Addressing this issue is a priority that would significantly impact the future of M&E of PA and would be a major facilitator for scaling up successful programs and data continuity. A study on the characteristics of PA initiatives conducted in Saudi Arabia found that all the initiatives have common attributes, such as, short-term initiatives, and need a coordinating body. Also, the majority of the PA initiatives needed objective evaluations of their outcomes [22].

#### Interest from multiple institutions in physical activity, with different definitions and indicators

A clear strength of Saudi Arabia is the diversity of institutions and stakeholders interested in PA. However, there is a need to unify how each institution defines PA. An attendee mentioned its organization’s experience in modifying and aligning how they defined and measured PA, with the organization shifting to using the standard WHO definition, unifying this definition with their partners, and conducting annual surveys to capture the levels of PA among the population since 2015. The GASTAT also conducts annual surveys to measure PA in the population [16].

Another example of a larger-scale attempt to unify efforts is the Gulf Center for Disease Prevention and Control’s (GCDC) strategy to develop a unified regional tool to collect data on PA levels in the region [23]. Each of the six Gulf Cooperation Council (GCC) countries– Bahrain, Saudi Arabia, Kuwait, Oman, Qatar, and the United Arab Emirates– currently has its separate national survey for data collection. The goal is to have a standardized survey to collect data that could be easily compiled and compared across the region. This is inspired by the smoking and tobacco survey in the Gulf area; each country updated its survey methodology, and they now have one unified survey.

To ensure comprehensive PA policy evaluation, upstream indicators such as policy and environment must be aligned with downstream indicators (individual determinants and behavior outcomes). Incorporating both into M&E and surveillance systems indicates that the information collected have the potential to signify real change, showing some population-level shifts towards healthy behaviors [12]. According to the WHO Global Action Plan on PA 2018–2030 [13], there is no single policy solution. In fact, increasing PA requires a systems-based approach with a strategic combination of upstream policy actions intended to improve the cultural, social, economic, and environmental determinants that support PA, in combination with downstream, focusing on educational and informational approaches [13].

#### Buy-in from leadership, with a need for a clear governance body

The stakeholders’ representatives drew on their experiences of the recent buy-in from leadership for making changes and improving public health in Saudi Arabia [10]. They reflected on the interest in PA from the government and the need for policies that support implementation. Nourah Health, for example, mentioned that one of the facilitators of success for their cohort study was the buy-in from the leadership of the university itself. An attendee noted that with strong policies that enforce the M&E of PA and communicate PA as a priority for the public through such strong policies, the funding streams would soon follow. Several attendees drew comparisons with the successful tobacco control programming in the country, which was facilitated by a central governance structure that represented and coordinated efforts across multiple sectors.

### Prominent recommendations to address high priority gaps

#### Establishing a multi-sectoral and specialized governance structure

It is important to examine the feasibility of establishing a strategy council or government body with representatives from different sectors. This council could sit under the MoH but needs to include all relevant stakeholders from different sectors and would undertake the planning, need assessments, and decision-making and provide technical support for implementing bodies working on PA in Saudi Arabia. The council would also oversee grants and other potential funding sources to support programs financially. Another essential function for the council could be to aid in unifying definitions of PA and the indicators used in M&E as well as the data collected by stakeholders within Saudi Arabia, while maintaining alignment with international definitions and indicators, such as those recommended by the WHO. The need for governance specific to PA has been iterated in the policy review by AlMarzooqi et al. [11] and in the workshop discussions. This can be considered the starting point for implementation of other recommendations.

### Developing a framework for engagement with different stakeholders

Developing a framework for engagement would enable regular conversations between stakeholders and the government, allowing all sides to hold each other accountable for their shared goals. The shared responsibility could catalyze the progression towards embedding M&E in the relevant PA-related programs. The framework should also include principles of engagement and indicators that can be evaluated to assess engagement quality. A good example of assessing the quality of stakeholder engagement is an analytical framework published by the United Nations Department of Economic and Social Affairs and the United Nations Development Program, which names inclusion, participation, and accountability as principles, each with their sub-indicators, which are all rated on 4 levels (level 0 to level 3) according to how strongly they are fulfilled [24].

### Establishing physical activity surveillance systems

It is well acknowledged that physical inactivity is a complex problem, influenced by multiple and interrelated drivers that require a “systems approach”, involving actions across multiple sectors [13, 14]. Surveillance systems for PA should be developed beyond the measurement of individual behaviors to monitor the wider sociocultural, environmental, and policy determinants of PA [22].

Surveillance is a core function of public health. Evaluating public health surveillance systems is paramount to properly monitor critical and significant public health events. The GCDC offers a good example of regional surveillance systems. It appears that the GCC surveillance systems are achieving optimal performance and showing favorable outcomes [25]. The stakeholder representatives agreed that incorporating PA monitoring and surveillance into the existing national or regional surveillance systems appears to be a preferred choice.

On an international level, Canada for example, has maintained a surveillance system encompassing periodic surveys of schools and workplaces, inspections of the built environment, and assessments of the social and economic components of PA [26]. In addition, routine PA surveys include objective pedometer measurements of representative samples of the population. The Canadian system, which meets key stakeholder needs, appears flexible and responsive to emerging priorities. In general, the Canadian experience shows that a complex and multi-sectoral PA surveillance system is feasible for informing PA policy [26]. The National PA Plan Alliance, the National PA Society, and the National Coalition for Promoting PA merged to form the foundation for the PA Alliance, representing a broadly based, powerful voice for PA promotion in the United States [27]. According to its description, “the Alliance combines deep expertise in policy advocacy, strategic planning, and workforce development to address PA”. They connect planning to policy advocacy and professionals involved in promoting public health approaches to PA [27]. In Australia, surveillance of PA components is collected by states and territories using their own data systems, methods, and indicators. However, the Australian Systems Approach to PA is a national project to develop systems approaches to PA, supported by the Australian Government Medical Research Future Fund [28].

### Developing a framework and guidance note for the evaluation of physical activity

During the conception of this workshop, stakeholders were interested in developing a context-specific guidance note to promote and facilitate high-quality evaluations of programs targeting PA. This guidance note is intended to be used by any stakeholder implementing a PA program or developing new PA programs to facilitate incorporating standardized evaluation methods into existing and new programs. Based on the workshop’s discussion and addressing the need for unified guidance, having a guiding framework will facilitate a more systematic approach to evaluation, as well as a shared understanding of the process between all involved stakeholders.

Through the lens of the evaluation framework, a standardized national evaluation approach could be developed (linked to a national strategy on PA), as well as additional evaluations, which could target smaller, more specific programs/population groups (e.g., school students or elderly) and also be done more frequently if needed. This guidance will also help ensure that a robust evaluation design is included as standard practice in program proposals, supporting knowledge generation, evidence-based implementation, and promoting accountability.

### Developing mechanisms for sharing experiences and knowledge

Overall, the workshop allowed attendees to engage in conversation, share knowledge of their work, learn about different initiatives by other organizations, and exchange contact information to continue discussion and collaboration. Dissemination of knowledge and creating new opportunities for collaboration is a strong desire of all attendees, and thus, more conferences, accessible online knowledge hubs, publishing of all relevant data, and organizing follow-up workshops or a technical working group will ensure the conversations, collaboration and initiatives continue to develop.

### Strengths and limitations

The strengths of this stakeholder perspective-gathering process lie mainly in building on existing capacities in Saudi Arabia. The discussion was held between a large group of key national stakeholders, who all have projects related to different aspects of PA. The conversation drew on their experiences and focused on learning from local scalable projects as well as regional and international guidelines and examples.

The approach had some limitations worth mentioning. While attendance and representation of the attendees was wide and most of the invited individuals were present, further key stakeholders were needed. Although this workshop is a great first step towards creating a national dialogue around PA, ongoing intentional dialogue is needed, especially with high-ranking decision-makers. Additionally, the workshop did not discuss M&E of PA and sedentary behaviors in workplaces. Given the large proportion of time many adults spend at work, M&E of PA in this setting represents a significant need. Finally, neither attendees nor their organizations were contacted after the workshop for additional verification of data. We also did not attempt to contact the small number of invited individuals who chose not to attend the event.

## Conclusion

The M&E of PA in Saudi Arabia is a subject that requires a multi-stakeholder, multi-sectoral approach that would be facilitated by the creation of a specialized governance and leadership committee, which could promote actions toward addressing additional important steps like creating a national strategy and action plan for PA, with an M&E framework that includes key goals, targets, indicators, unified data collection methods, and guidance on how to ensure coordinated and wide knowledge dissemination. Involving additional stakeholders in the follow-up events and conversations for a stronger discussion, greater inclusivity of experiences, and establishing links with additional stakeholders. Policymakers need to embrace such an agenda and advance the discussions on the scope and structure of M&E and surveillance system for PA.

## Electronic supplementary material

Below is the link to the electronic supplementary material.

Supplementary Material 1: Additional file 1. Workshop program

Supplementary Material 2: Additional file 2. Summary of discussions in breakout sessions

Supplementary Material 3: Additional file 3. List of stakeholders identified by the workshop participants

## Data Availability

No datasets were generated or analysed during the current study.

## Abbreviations

GASTAT: General Authority for Statistics
GCC: Gulf Cooperation Council
GCDC: Gulf Center for Disease Prevention and Control’s
KFSHRC: King Faisal Specialist Hospital and Research Center
M&E: monitoring and evaluation
MoH: Ministry of Health
NC: Neighborhood Clubs
NCDs: Noncommunicable diseases
PA: physical activity
SFA: Sports for All Federation
WHO: World Health Organization
WHS-SA: World Health Survey-Saudi Arabia
