# Supplementary material for: Stakeholder perspectives on surveillance of physical activity and monitoring and evaluation of interventions in Saudi Arabia
Source: BMC Public Health. 2025 Apr 12;25:1381. doi: 10.1186/s12889-025-22631-5 (PMC11992715; doi:10.1186/s12889-025-22631-5)
Supplement: Supplementary file 1 — Supplementary Material 1: Additional file 1. Workshop program [file 12889_2025_22631_MOESM1_ESM.docx]

**Additional file 1: Workshop program**

|  | **Content** | **Duration** |
| --- | --- | --- |
|  | **Opening remarks and introductions** | 09:00am - 09:20am |
|  | **Objectives and agenda of the workshop** | 09:20am - 09:40am |
|  | **Setting the stage: Physical activity in Saudi Arabia**  **Questions and answers** | 09:40am - 10:00am |
|  | **Break + Transitioning to small groups** | 10:00am – 10:05am |
|  | **Breakout: Small group discussion**  *Breakout 1: What is the current status of physical activity surveillance and monitoring and evaluation in Saudi Arabia*   - *Attendees to split into small groups* - *Facilitator to guide the discussion and obtain a volunteer from the group to be the group representative for the large discussion*   *End goal of identifying key successes and challenges in physical activity surveillance and monitoring and evaluation in Saudi Arabia* | 10:05am – 11:00am |
|  | **Plenary: Report back and large group discussion**   - *Small group representatives to take turns reporting back* - *Compiling and identifying the points of alignment between all small groups*   Questions and answers | 11:00am – 12:00pm |
|  | Prayer break (Duhr) + Lunch | 12:00am – 01:00pm |
|  | **Small group discussion**  *Breakout 2: Priority actions and stakeholders to strengthen physical activity surveillance and monitoring and evaluation*   - *Attendees to split into small groups* - *Facilitator to guide the discussion and obtain a volunteer from the group to be the group representative for the large discussion*   *End goal of identifying priority actions and the relevant stakeholders to strengthen physical activity surveillance and monitoring and evaluation in Saudi Arabia* | 01:00pm – 02:30pm |
|  | **Plenary: Report back and large group discussion**   - *Small group representatives to take turns sharing their list of priorities and actions* - *Compiling and identifying the points of alignment between all small groups*   *Questions and answers* | 02:30pm – 03:15pm |
|  | **Coffee break** | 03:15pm – 03:30pm |
|  | Next steps and closing remarks   - *Summarizing the compiled list* - *Thanking the attendees* - *Highlighting next steps*   *Closing remarks* | 3:30pm – 4:00pm |
|  | END OF WORKSHOP | 4:00pm |
