# Supplementary material for: Stakeholder perspectives on surveillance of physical activity and monitoring and evaluation of interventions in Saudi Arabia
Source: BMC Public Health. 2025 Apr 12;25:1381. doi: 10.1186/s12889-025-22631-5 (PMC11992715; doi:10.1186/s12889-025-22631-5)
Supplement: Supplementary file 2 — Supplementary Material 2: Additional file 2. Summary of discussions in breakout sessions [file 12889_2025_22631_MOESM2_ESM.docx]

**Additional file 2: Summary of discussions in breakout sessions**

| **Session** | **Group** | **Summary of discussion per group** |
| --- | --- | --- |
| Breakout session #1:  What is currently working and not working in physical activity (PA) surveillance and monitoring and evaluation (M&E) in SA? | Group 1 | Current Status:   1. Strong programs/Initiatives (However, individualized efforts) 2. There are success stories 3. Buy-in from the leadership (Vision 2030, Vibrant Society) 4. Collaboration with global entities (e.g., World Bank Group, World Health Organization) 5. Accessibility of physical activity for communities (Available platforms) 6. Readiness of the community   Current Challenges:   1. The need for diverse stakeholder engagement 2. The need for a centralized governing body 3. The need for an evaluation/framework 4. The need for a standardized methodologies 5. The need for a guidelines for the evaluation of PA 6. Educational PA programs (Especially in schools in the early years) 7. Simple language (Education/Awareness to the public) 8. The need for more professionals in physical education 9. The need to utilization of technology (social media, artificial intelligence?) |
|  | Group 2 | Current Status:   1. Adaptations of new innovative techniques and approaches across the board, for example, experience maps, hosting global events, mall activations, measuring through wearables and apps 2. The focus is collecting precise data and insights from multiple entities to entertain geographical cuts/slices, age groups, and interests.   Current Challenges:   1. The need for systematic data collection and impact evaluation tools and efforts 2. The need for institutional surveillance standards and criteria, for example, characteristics of targeted profiles of participants 3. Unify the broader definition of physical activity vs sports, resulting in either mismatched outcomes or duplicated efforts 4. Currently, most PA participation incentives are cost-driven, which may be effective today; however, sustaining results in the longer term may be less effective. 5. The need of a centralized knowledge hub to reference new trends, research, lessons learned, or insights related to health-promoting and PA |
|  | Group 3 | Current Status:   1. Monthly surveys (Saudi Sports for All Federation) 2. Technical sectoral committees 3. Health in All Policies 4. Many assets in Riyadh can be used to incentivize PA and can be evaluated.   Current Challenges:   1. The need for governance body 2. Need for differentiated approaches by population groups. 3. More need for evidence/ local research from Saudi Arabia 4. The need to unify – different surveys, metrics, goals 5. Increase population awareness of their own health 6. Data is focused on outputs, not outcomes 7. The need for in-depth research on how to apply learning from research into implementation 8. The need to disseminate more evidence 9. Data quality uncertain – e.g. data on PA among school-aged children 10. Lots of small initiatives – difficult to evaluate all as not enough capacity and small samples 11. Need for a long-term cohort with different population groups 12. Need for approaches/ policies by setting 13. Difficult to track longer-term outcomes (e.g., diabetes control). 14. Need to include patient-reported outcomes (e.g., quality of life) 15. Need for more qualitative research to understand the barriers 16. Health sector need to more focus on lifestyle/ prevention |
| Breakout session #2:  Priority actions and stakeholders to strengthen PA surveillance and M&E, including strategies to support collaboration | Group 1 | Priority actions:   1. Establishing a national coalition of physical activity. Multi-sectoral – perhaps reports to the Ministry of Health / Quality-of-Life Program 2. Establishing stable funding streams to support M&E programs 3. Identifying indicators specific to PA, starting with indicators that are established at the national level. 4. Creating a larger scale NCD risk factor survey including PA in addition to PA specific surveys 5. Focusing on healthy settings (whether the setting is a school, an employer, or a mall) 6. Investing in understanding the determinants of health that govern PA (including weather, terrain, and urban planning)   Examples of stakeholders:   1. Everyone can be a stakeholder. The more we are involved, the better our chances of success. 2. Public Health Authority – part of the governing model 3. Quality of life program as an empowering body |
|  | Group 2 | Priority actions:   1. Establishing a national coalition of physical activity. Multi-sectoral – perhaps reports to the Ministry of Health / Quality-of-Life Program 2. Establishing stable funding streams to support M&E programs 3. Identifying indicators specific to PA, starting with indicators that are established at the national level. 4. Creating a larger scale NCD risk factor survey including PA in addition to PA specific surveys 5. Focusing on healthy settings (whether the setting is a school, an employer, or a mall) 6. Investing in understanding the determinants of health that govern PA (including weather, terrain, and urban planning)   Examples of stakeholders:   1. Private sector 2. General Authority for Statistics 3. Media (Quality of Life Program) 4. Education sector 5. Universities and academic/research institutions 6. Ministry of Municipal and Rural Affairs and Housing 7. Giga project (NEOM) 8. Ministry of Labor 9. Ministry of Investment 10. Ministry of Transport 11. Ministry of Environment, Water, and Agriculture 12. Ministry of Interior 13. Ministry of Tourism 14. Ministry of Commerce 15. Mahd academy 16. Islamic affairs 17. Nongovernmental organizations 18. International organizations (World Health Organization, World Bank, other agencies of the United Nations) 19. Ministry of Finance 20. Ministry of Foreign Affairs 21. Council of Health Insurance/insurance companies |
|  | Group 3 | Priority actions:   1. National framework for M&E for PA 2. Promote specific evaluations of innovations, such as incentives (with Council of Health Insurance; e.g., SAFEWAY model, insurance companies and cars), Royal Commission for Riyadh City SPL prototype 3. Develop guidelines/ policies to target private sector to incentivize PA promotion and showcase evidence (e.g., Cleveland clinic as an example of organizational promotion of PA among employees), but there needs to be policies/ guidelines to encourage private sector to initiate similar programs 4. Establish national surveillance system 5. Online dashboard showcasing progress in PA in SA 6. Promote qualitative research 7. Explore opportunity of having a “super app” as a single source for reporting PA (e.g. lessons from organ donation, COVID-19 vaccine) 8. Explore innovative data/ M&E system e.g. Sports Boulevard 9. Quality of Life Program – need to leverage this platform to promote collaboration 10. Annual conference for PA in SA 11. Peer groups (e.g. elderly) 12. Code areas for global positioning system (e.g., gym, park, green places) – for walkability   Examples of stakeholders:   1. All stakeholders represented here PLUS 2. Nongovernmental organizations 3. Civil society (groups and individuals) 4. Ministry of Education 5. Health Holding Company 6. Council of Health Insurance 7. Private sector 8. Ministry of Municipal Affairs 9. Academic Institutes 10. Ministry of Economy and Planning 11. NEOM, Sports Boulevard and other mega projects 12. Ministry of Tourism 13. Health Sector Transformation Program |
