# Supplementary material for: Stakeholder perspectives on surveillance of physical activity and monitoring and evaluation of interventions in Saudi Arabia
Source: BMC Public Health. 2025 Apr 12;25:1381. doi: 10.1186/s12889-025-22631-5 (PMC11992715; doi:10.1186/s12889-025-22631-5)
Supplement: Supplementary file 3 — Supplementary Material 3: Additional file 3. List of stakeholders identified by the workshop participants [file 12889_2025_22631_MOESM3_ESM.docx]

**Additional file 3: List of stakeholders identified by the workshop participants**

1. Government and Public sector
   1. Ministry of Human Resources and Social Development
   2. Ministry of Investment
   3. Ministry of Transport and Logistics Services
   4. Ministry of Environment, Water, and Agriculture
   5. Ministry of Interior
   6. Ministry of Tourism
   7. Ministry of Commerce
   8. Ministry of Education
   9. Ministry of Islamic Affairs, Dawah, and Guidance
   10. Ministry of Municipal and Rural Affairs and Housing
   11. Ministry of Finance
   12. Ministry of Foreign Affairs
   13. The National Center for Performance Measurement
   14. Health Sector Transformation Program
   15. Quality of Life Program
   16. Mahd sports academy
   17. General Authority for Statistics
   18. Council of Cooperative Health Insurance
2. The Private sector
   1. Health holding companies
   2. Mega/Giga projects (e.g. NEOM, the New Riyadh’s Downtown)
3. Nongovernmental organizations
4. Universities and academic/research institutions
5. International organizations (World Health Organization, World Bank, and other agencies of the United Nations)
6. Civil society (groups and individuals)
